# Valeo Stent Use for Ductal-Dependent Systemic Circulations

**DOI:** 10.1007/s00246-025-03931-4

**Published:** 2025-06-26

**Authors:** Mayank Sharma, Kamel Shibbani, Mohamed Khallaf, Bassel Mohammad Nijres, Osamah Aldoss

**Affiliations:** 1 Division of Pediatric Cardiology, Stead Family Children’s Hospital, University of Iowa, 200 Hawkins Dr, BT-1010, Iowa City, IA 52241 USA; 2 Division of Pediatric Cardiology, Icahn School of Medicine at Mount Sinai, New York City, NY USA; 3 Pediatric Cardiology, King Faisal Specialist Hospital and Research Center, Jeddah, Saudi Arabia; 4 Department of Pediatrics, Division of Cardiology, Medical College of Wisconsin, Children’s Wisconsin, Herma Heart Institute, Milwaukee, WI USA

**Keywords:** Ductal stenting, Valeo, Ductal dependent systemic circulation, Hybrid

## Abstract

Our aim is to describe the experience of ductal stenting using the balloon-expandable Valeo stent in ductal-dependent systemic circulation. Outcomes assessed were procedural success, procedural survival, stent related complications, and re-intervention rates. Hybrid palliation, consisting of ductal stenting and bilateral pulmonary artery band placement, is a widely accepted alternative for stage I palliative surgery. Off-label use of stents is the norm for this procedure. The Valeo stent is one such example with minimal data on its use for ductal stenting. Retrospective data was collected for seventeen patients who underwent the hybrid procedure with ductus arteriosus stenting and pulmonary artery banding between November 2019 and September 2024. Descriptive statistics were performed. Seventeen patients underwent the hybrid procedure between November 2019 and September 2024 with a median age of 14 days (IQR 10–20 days) and median weight of 3.47 kg (IQR 3.2–4.2 kg). Fifteen patients had a single stent implanted at the time of the initial procedure and two patients required an additional stent. There were no intraoperative deaths, there was one in-hospital death. The interstage mortality was 30%, 12 out of 17 patients survived to subsequent planned procedure. The median duration to subsequent palliation was 157 days, during which no patients developed any in-stent stenosis or stent migration. The re-intervention rate was 35%. This consisted of 5 patients that required additional stents in the inter-stage period to address the ductal constriction in the pulmonary or aortic end and 1 patient that required balloon angioplasty for somatic growth. The Valeo stent can be a suitable option for ductal stenting during hybrid procedure in patients with hypoplastic left heart syndrome and other left heart obstructive lesions. It is an effective stent for maintaining ductal patency with good safety profile and a low rate of in-stent stenosis due to intimal proliferation during the inter-stage period.

## Introduction

Since its first introduction in the 1990s as a less invasive alternative to the Norwood surgery, the hybrid procedure has become a common procedure across centers for the management of patients with hypoplastic left heart syndrome (HLHS) and its variants [1–5]. The hybrid procedure consists of bilateral pulmonary artery banding and ductal stenting. Centers vary in their approach with some performing bilateral pulmonary artery (PA) banding followed by ductal stenting in two separate procedures (Giessen approach) and others performing them in the same setting through direct transpulmonary access (Columbus approach) [4–8].

Stent selection for ductal stenting is dependent on many factors including desired stent length, stent design, stent availability, and operator preference. While initial stents used for ductal stenting during hybrid procedures were balloon expandable, recent years have seen the use of self-expandable stents as well [2, 3, 7]. The ideal patent ductus arteriosus (PDA) stent should have an appropriate radial force, should be amenable to delivery through a small sheath, and should be able to conform to the anatomy of the ductus. Because this ideal stent does not exist, interventional cardiologists have had to rely on off-label use of various types of stents, with coronary artery stents and peripheral vascular stents being commonly used [7, 9].

The Bard Valeo™ peripheral vascular stent (Bard Peripheral Vascular, Tempe, Arizona, United States of America) is a triple-helical architecture stent with an open-cell design that comes pre-mounted on low-profile balloons. Though originally designed as a biliary stent, the Valeo stent has been utilized in pediatric congenital heart disease and pediatric vascular interventions including ductal stenting [10–14].

Although the initial application of the hybrid procedure has been described largely in patients with the HLHS, its application has been extended to encompass all lesions with ductal-dependent systemic circulation [8, 15–17]. The aim of the present study is to evaluate safety and efficacy of using the Valeo stent for PDA stenting in the setting of ductal-dependent systemic circulation. This includes HLHS, interrupted aortic arch (IAA), hypoplastic arch, and aortic atresia.

## Materials and Methods

We performed a retrospective data review to assess feasibility and safety of the use of Bard Valeo ™ stents to maintain ductal patency in HLHS and various other left heart obstructive lesions with ductal-dependent systemic circulation. We included all patients with hypoplastic left heart syndrome, hypoplastic left heart complex, interrupted aortic arch, hypoplastic arch, and aortic atresia who underwent ductal stenting using a Valeo stent at the University of Iowa Hospitals between November 2019 and September 2024. The study was approved by our institutional review board. Patient characteristics collected included gestational age at birth, cardiac diagnosis, age at intervention, and body weight at the time of intervention. Procedural characteristics collected included total duration of the procedure, total duration of fluoroscopic exposure and total radiation dose (indexed and non-indexed), access site used for PDA stent delivery, any additional interventions done aside from PDA stenting, and complications during the procedure. Procedural time was defined as the time between sheath-in and sheath-out. Re-intervention included all instances where cardiac catheterization was performed after the index procedure and an intervention took place, whether these were planned or unplanned. This included any intervention done to address in-stent stenosis, stent fracture or deformation, and stent migration. It also included any intervention to balloon the take-off of the jailed retro-aortic arch or the addition of another stent to ensure coverage of the entire duct. This also included balloon angioplasty to account for somatic growth. The primary outcomes were freedom from mortality during the procedure, success of PDA stenting, and freedom from mortality in the inter-stage period. Successful PDA stenting was defined as a patient leaving the catheterization laboratory alive with the PDA stent in place on final angiogram. Secondary outcomes included incidence of stent fracture and rates of re-intervention before the subsequent surgery. Descriptive analysis was performed on the total population.

### Procedure Description

All stent implantations were performed under general anesthesia in a biplane cardiac catheterization laboratory. For the transfemoral approach, surgical pulmonary artery banding was done 2 days – 2 weeks prior to PDA stenting. At the time of PDA stenting, angiograms were obtained using pigtail catheter. An end-hole catheter was used to cross the PDA from the femoral venous side, and a 0.035″ guidewire was advanced into the descending aorta. The short femoral sheath was exchanged for a long sheath (6–7 Fr Brite Tip™, Cordis) over the 0.035″ wire. The Valeo stent diameter was chosen such that it is oversized by 1–2 mm to the narrowest diameter of the duct. The Valeo stent was then deployed with balloon inflation to pressures ranging from 8 to 16 ATM. Angiograms were obtained to confirm stent positioning. Prostaglandin infusion was stopped after deployment of the stent while the patient was still in the catheterization lab.

There was one instance where the artery was used for retrograde cannulation of the duct in the setting of an interrupted IVC. The PDA was crossed in a retrograde fashion from the descending aorta. Angiograms were performed through a pigtail catheter positioned in the PDA. A 0.035″ was advanced into the ventricle and used to exchange the short sheath for a 7 Fr long sheath that was used to deploy the stent.

For the transpulmonary approach, a sternotomy was performed for bilateral pulmonary artery banding. Access into the MPA was obtained by the surgeon and a long sheath (Brite Tip™, Cordis) was advanced into distal MPA. Angiography was performed by direct injection through the long sheath. A 0.035″ guidewire was advanced into the descending aorta and used to deliver the Valeo stent as described above.

## Results

### Patient Characteristics

Between November 2019 and September 2024, seventeen patients underwent the hybrid procedure with ductal stenting and PA banding. Nine patients (53%) had single ventricle circulation, while 8 patients (47%) had biventricular circulation (Table 1). Of the 9 patients with single ventricle circulation, 6 patients had hypoplastic left heart syndrome (HLHS), 1 had double inlet left ventricle with hypoplastic right ventricle, malposed great vessels and hypoplastic arch, 1 had right ventricle dominant unbalanced AV Canal defect with hypoplastic arch, and 1 had hypoplastic arch with small mitral and aortic valves and a borderline LV that was deemed to be more suitable for a single ventricle. The median gestational age at birth was 39 weeks 2 days and the median birth weight was 3196 g. The median age at the time of first PDA stent implantation was 14 days (IQR 10–20 days). The oldest patient was 127 days old at the time of the procedure. In this patient with HLHS and heterotaxy, ductal stenting was aborted at 24 days of life due to ST segment changes during the diagnostic portion of cardiac catheterization. Subsequently the patient was deemed to be a transplant candidate but underwent PDA stenting due to persistent desaturation while awaiting transplant. The median weight at the time of first stent implantation was 3.47 kg (IQR 3.2 -4.2 kg).

**Table 1:** Patient characteristics with diagnosis, age, body weight, and number of stents needed at index procedure

Transfemoral Access (Giessen approach)					
Patient Characteristics			Stent Size(s)	Inter-stage interventions	Outcome
Indication for ductal stent	Age (days)	Weight (Kg)			
HLHS (MS/AA)	17	3.2	8 × 26	No intervention	Interstage death due to bowel perforation, sepsis
HLHS (MS/AA)	18	4.4	9 × 26	Additional 9 × 17 mm Valeo stent in pulmonary end	Out of hospital death after Norwood BD Glenn
HLHS (MS/AS)	20	4.26	7 × 26	No intervention	Fontan
HLHS (MA/AA)	127	5.3	10 × 26, 10 × 26	No intervention	Death after heart transplant
HLHS (MS/AS)	7	4.2	8 × 26	Additional 8 × 18 mm Valeo stent in pulmonary end	Fontan
Hypoplastic arch, VSD	30	3.05	8 × 18	No intervention	Biventricular repair
IAA Type A	12	3.3	9 × 26	No intervention	Truncus arteriosus repair
IAA Type A	16	3.5	7 × 18	No intervention	IAA Repair

Transpulmonary Access (Columbus approach)					
Patient Characteristics			Stent Size(s)	Inter-stage interventions	Outcome
Indication for ductal stent	Age (days)	Weight (Kg)			
IAA type A	25	2.4	7 × 18	Additional 8 mm Valeo stent in aortic end, PDA stent ballooning	Out of hospital cardiac arrest after surgical repair
Hypoplastic arch, D-TGA	14	3.42	9 × 17	No interventions	Arterial switch
Hypoplastic arch, parachute MV, borderline LV	27	3.9	7 × 26	No interventions	Biventricular repair
HLHS (MS/AA)	0	3	9 × 17	No interventions	Interstage in-hospital death due to cardiac arrest
Hypoplastic arch, small MV, small LV	9	3.5	8 × 26	No interventions	Biventricular repair
Hypoplastic arch, DILV and hypoplastic RV	11	3.2	10 × 36	No interventions	Truncus arteriosus repair
IAA type B	10	3.4	10 × 17,10 × 17	Additional 9 × 17 mm Valeo stent in aortic end	IAA repair
Hypoplastic arch, unbalanced RV dominant AVCD	9	4.4	7X18	Additional 7 × 18 mm Valeo stent in pulmonary end	IAA repair
Aortic atresia, VSD	6	3.52	8 × 26	Balloon angioplasty of Valeo stent for somatic growth	Biventricular repair

Abbreviations: *PDA* Patent ductus arteriosus, *HLHS* Hypoplastic left heart syndrome, *MS* Mitral stenosis, *MA* Mitral atresia, *AA* Aortic atresia, *AS* Aortic stenosis, *MV* mitral valve, *RV* Right ventricle, *LV* Left ventricle, *AV* Atrioventricular canal defect, *IAA* Interrupted aortic arch, *D-TGA* Dextro transposition of great arteries

### Procedural Characteristics

The transfemoral approach (Giessen approach) was used in 8 patients, while the transpulmonary approach (Columbus approach) was used in 9 patients. The median procedure time was 71 min for the transfemoral approach and 139 min for the transpulmonary approach. The average prostaglandin infusion rate was 0.025 µg/kg/min. The median fluoroscopic exposure time for the transfemoral procedures was 14.2 min and the median Dose Area Product was 13.1 cGy-cm^2^. For the transpulmonary approach, the median fluoroscopic exposure time was 3.1 min and the median Dose Area Product was 3.7 cGy-cm^2^. Of the 8 patients who underwent transfemoral PDA stenting, the right femoral vein was used as the access site in 7 patients with a 6 Fr being the most used sheath size. The right femoral arterial access was used in a single instance in a patient who had heterotaxy with interrupted inferior vena cava (IVC). The patient was 127 days old and weighed 5.3 kg at the time of procedure. A 7 Fr sheath was used, and the patient received heparin infusion for 24 h without any subsequent femoral artery thrombosis. Of the nine patients who underwent the PDA stent and PA bands in a single procedure day, direct MPA access was used for PDA stent delivery in 8 patients. In one patient, the sheath was placed directly in the RVOT due to horizontal MPA orientation. For the transpulmonary approach, a 7 Fr sheath was the most frequently used sheath size.

Twenty-four Valeo stents were deployed in seventeen patients between November 2019 and September 2024. The stent size used ranged from 7 × 26 mm to 10 × 36 mm. The median number of stents deployed during the index procedure was 1. Two patients required two stents to be placed in the index procedure. The most used stents diameters were the 7 mm (n = 5) and 8 mm (n = 5), and most used stent length was 26 mm (n = 9).

Our experience with the hybrid procedure evolved over time, with the initial eight patients undergoing surgical PA banding followed by transfemoral PDA stent placement in two separate procedures (Giessen approach). The remaining 9 patients underwent a hybrid procedure where surgical PA banding was performed followed by direct transpulmonary PDA stenting done in the same procedure (Columbus approach). This variation in the hybrid approach was due to institutional experience, with a shift toward a single procedure as our institutional comfort level increased. Five patients underwent a balloon atrial septostomy before the hybrid procedure depending on their anatomy.

### Characteristics of the Duct

The average PDA length was 18.5 mm, and the average narrowest diameter was 6 mm, with an average increase of narrowest diameter by 2 mm after the PDA stent placement. Two patients required an additional stent during the first procedure. In the first patient, the stent slipped off the balloon several millimeters anteriorly during inflation resulting in under expansion of the anterior portion of the balloon and lack of appropriate coverage of the PDA posteriorly. On removal of the balloon, it was noted that the balloon had also ruptured during deployment. This required a second more distal stent to be placed. In the second patient, the PDA was about 3 cm in length and required two stents to traverse the entire length.

### Primary Outcomes

**Fig. 1:**
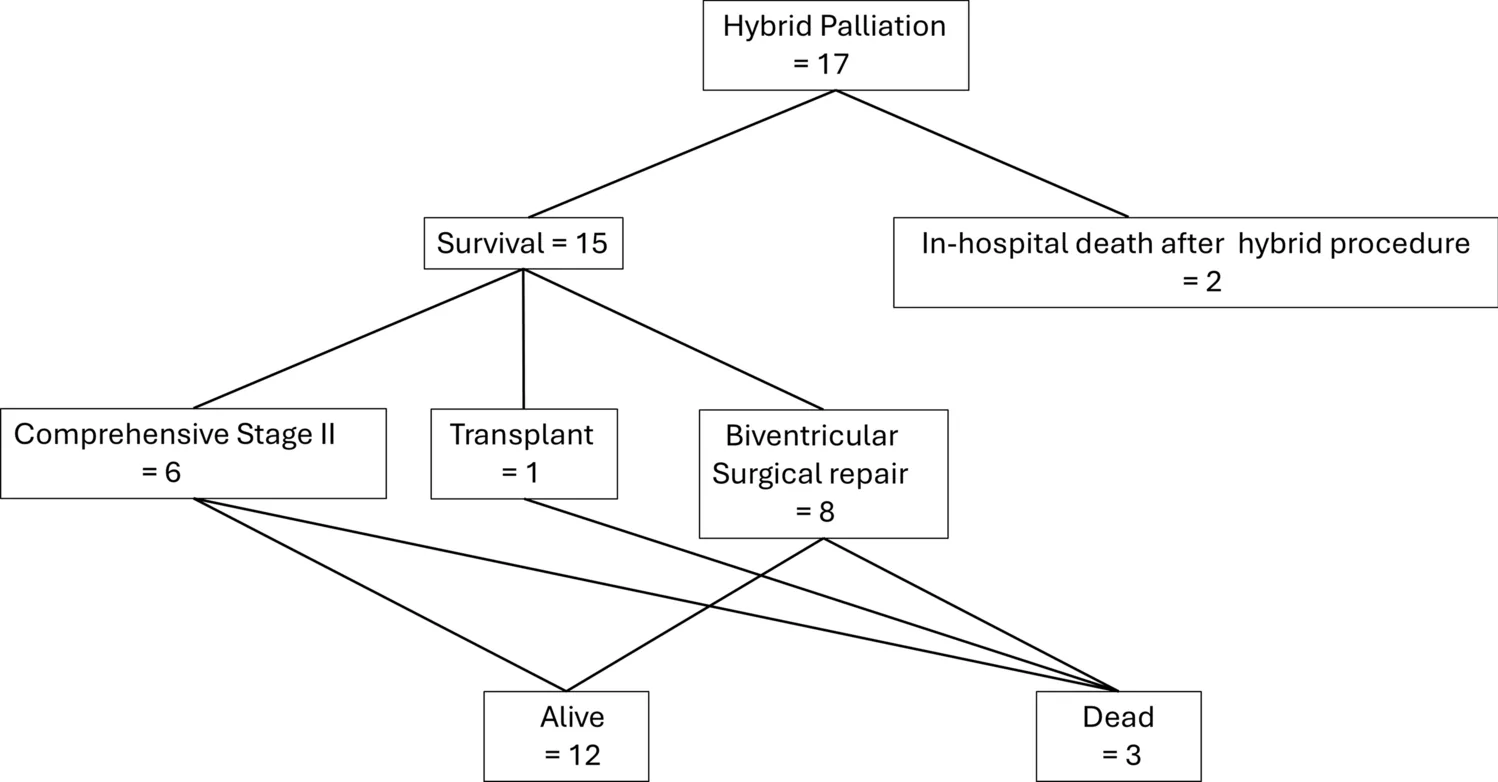
Outcomes after hybrid palliation. Figure shows total number of patients who underwent hybrid palliation and the overall outcomes

There were no mortalities reported during cardiac catheterization, with all patients successfully undergoing PDA stent placement. Interstage mortality was 12% (n = 2). Both patients had HLHS and died in the interstage period before the comprehensive second stage operation (Fig. 1). The first patient was on extracorporeal membrane oxygenator (ECMO) support prior to the index procedure. He decompensated 1 day after the procedure due to progressive right ventricular failure despite ECMO. Diagnostic catheterization done showed a well expanded PDA stent with no evidence of jailing of the retrograde arch. The patient decompensated further and had a sudden cardiac arrest with return of spontaneous circulation. A CT scan showed concerns for diffuse thrombosis, including thrombus in the ascending aorta, aortic arch, PDA stent, and descending aorta. No further interventions were performed due to extensive stent thrombosis and poor prognosis. The second patient expired in the PICU after a prolonged stay of 167 days due to co-morbidities of severe tracheomalacia and small bowel perforation. There were no stent related concerns at the time of death.

### Secondary Outcomes

One patient had a self-contained vascular injury in the anterior main pulmonary artery that did not require any intervention. This patient had the PDA stent implanted through femoral venous access. There was one incidence of balloon rupture during stent deployment, described above. 

A total of six patients (35%) required re-interventions on the stent for indications such as systemic pre- and post-ductal desaturations, increased gradients across the PA bands on echocardiogram, decreased biventricular function, acute cardiorespiratory decompensation or concern for ductal obstruction.

**Fig. 2:**
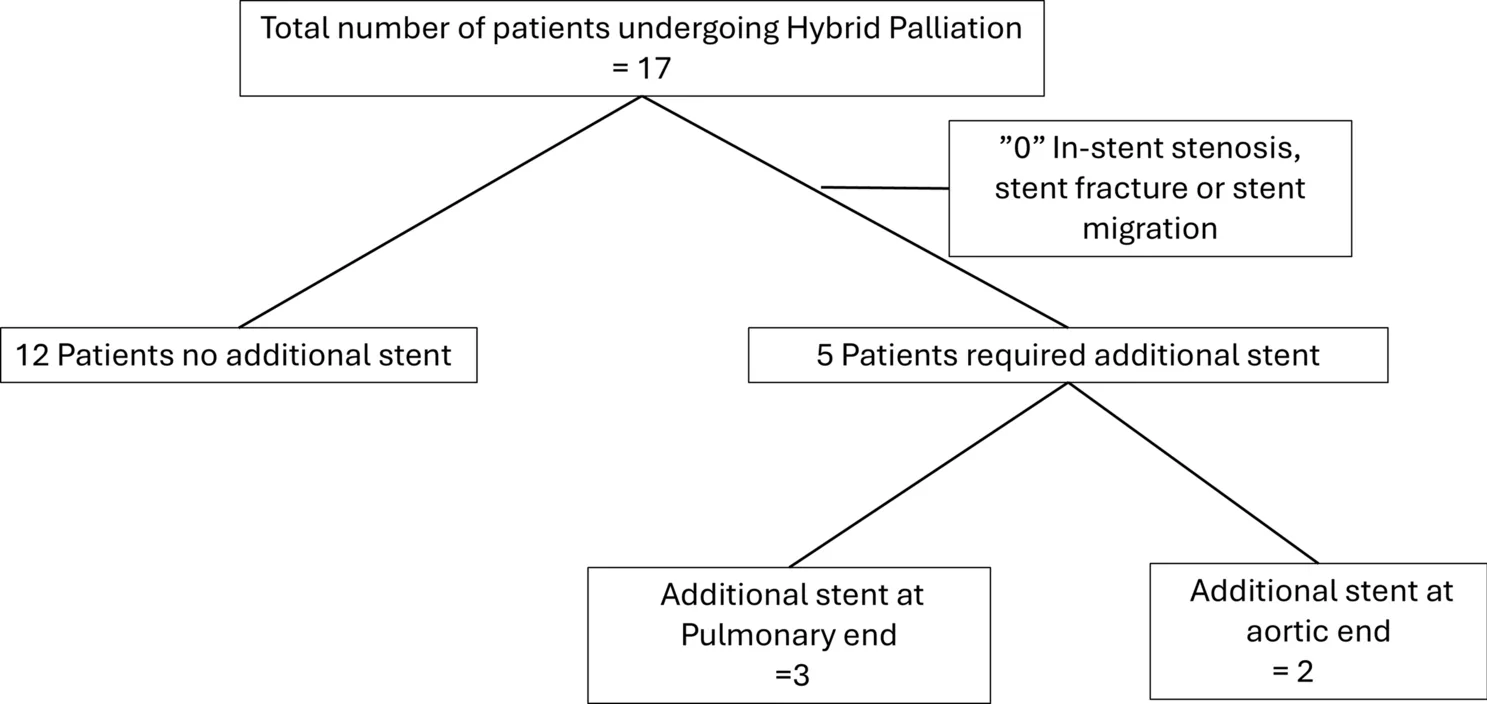
Valeo Stent performance after hybrid procedure. Figure shows stent performance during follow-up period with no stent stenosis, fracture or stent migration

Five patients required an additional Valeo stent placement in the ductus (Fig. 2). In three of these patients, ductal constriction was seen at the pulmonary end due to the original stent not covering the entire length of the PDA. Two patients required an additional Valeo stent in the PDA due to ductal narrowing seen at the aortic end. There was one patient with aortic atresia and VSD that required balloon angioplasty of the ductal stent on three different occasions to keep up with somatic growth while awaiting biventricular repair with Yasui procedure. There was a concern for jailed retrograde arch in this patient, so balloon angioplasty of the jailed descending aorta was performed through the side struts of the stent.

There were no instances of stent recoil, stent fracture, or significant in-stent stenosis noted on follow-up catheterization in any of the patients. One patient with Shone’s like complex had mild in-stent stenosis of the superior aspect of the PDA stent seen during pre-surgical diagnostic catheterization, which did not require any intervention.

## Discussion

Herein, we describe our experience of using the Valeo stent for PDA stenting in hybrid procedure for ductal-dependent systemic circulation. The Valeo stent is a balloon-expandable bare-metal triple-helix open-cell stent that is pre-mounted on a low-profile nylon balloon. The pre-mounted stent is deployed over a 0.035-inch guide wire. It is available in diameters ranging from 6 to 10 mm in 1 mm increments, and in lengths from 17 and 56 mm. The 6–8 mm stents can be delivered through a 6 Fr sheath, while a 7 Fr sheath is needed for the 9–10 mm stents. The open-cell design allows for post-dilation with minimal sent foreshortening. The 9 and 10 mm Valeo stents can be taken up to 20 mm diameters, whereas the 6–8 mm diameter stents can be dilated up to 13 mm [11].

Our hybrid approach for PDA stenting was an evolving strategy that started out as a two-step procedure (Giessen-like hybrid approach) and transitioned to a single procedure (Columbus approach) as described by Galantowicz et al. in their landmark publication. Institutional experience at the beginning of the study period dictated our approach with initial surgical pulmonary banding, followed shortly by transfemoral PDA stenting a second procedure. As our comfort level increased, we moved toward a single combined hybrid procedure where pulmonary artery bands are surgically placed, and the surgeons provide direct MPA access for PDA stent placement. This shift was also precipitated by one case, described earlier, of a self-contained vascular injury to the MPA during sheath advancement from the transfemoral approach. The benefits of the single procedure are multiple and include reducing the number of anesthetic procedures, avoiding crossing the tricuspid and pulmonary valves with a sheath and therefore preserving the integrity of these valves, shorter runs of prostaglandin infusion since PDA stenting can be performed quicker and is no longer staggered with pulmonary artery banding, and having the surgeons physically present in the room for improved collaboration and bail-out if needed. Septostomy was not routinely done as not all patients required this procedure, but when it was needed, it was generally done before ductal stenting.

**Fig. 3:**
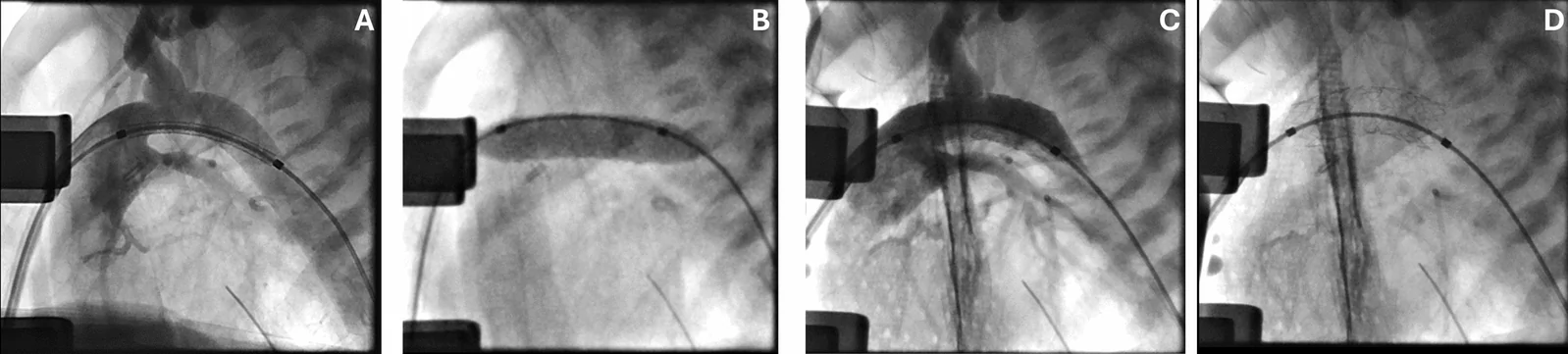
Example deployment of Valeo stent demonstrating flexibility of the stent. Patient with atretic aortic valve, severely hypoplastic ascending aorta and proximal transverse arch, and a large PDA. The ductus before the take-off of the retrograde arch was short and deploying a stent to cover only that part was felt to be risky with concerns of missing ductal tissue. Therefore, the decision was made to use a longer stent and extended into the descending aorta to ensure coverage of the entire PDA**.** A – Initial angiography shows a short PDA. The banded pulmonary arteries are noted. Retractors can be seen from the surgical PA banding. This was a Hybrid procedure (Columbus approach) with transpulmonary access. B – Deployment of the Valeo 8 mm × 26 mm stent. The anatomy conform to the balloon. C – Post-deployment angiography shows that the stent readily conforms to the anatomy of the PDA and descending aorta with minimal deformation. D – Same angiogram showing the stent without contrast

There is currently limited experience of using the Valeo stent in congenital heart disease and its use is entirely off label [12, 18, 19]. However, the Valeo stent offers several benefits for use in pediatric patients in general and for ductal stenting in particular, especially in lesions that have a ductal-dependent systemic circulation. First, the open-cell design allows for easier access to jailed vessels with bench testing showing that the side-cells of the Valeo stent can be maximally dilated up to 12 mm [11]. This is important during stenting of a lesion with ductal-dependent systemic circulation as the head and neck vessels are prone to being jailed by the stent. Second, the Valeo stent has minimal foreshortening during deployment [11, 20], which makes it a good choice for PDA stenting where missing ductal tissue can result in acute and profound decompensation. Third, the increased flexibility of the Valeo stent means that it can more readily conform to the anatomy (Fig. 3) [10]. One of the issues that can limit the use of Valeo stents in congenital heart disease lesions is the low radial force that the Valeo stent provides, though this is less relevant in instances of ductal-dependent systemic circulation where the lower radial strength is more than sufficient to maintain ductal patency.

Aside from the above-mentioned characteristics of the Valeo stent that make it a suitable option for PDA stenting in ductal-dependent systemic circulation, the Valeo stent was the stent of choice in our center for multiple reasons. At the beginning of our experience, we did not readily have access to self-expanding stents, and therefore relied on balloon expandable options. Second, we felt that the Valeo stent, with its range of diameters and lengths, provided appropriate sizing options for our population. Third, it was a stent that our team had used and was familiar with. Fourth, even when self-expanding stents were readily available, we opted to continue using balloon-expandable stents because of their propensity for post-deployment dilation, which is more limited with self-expanding stents. Our experience confirms the safety and efficacy of the Valeo stent for ductal-dependent PDA stenting. On initial implantation, the Valeo stents did not require post-deployment balloon dilation and on follow-up catheterizations the stents were found to be widely patent without in-stent stenosis or recurrent ductal constrictions. The cases of additional stent deployment after initial procedure were to address the ductal tissue that was not covered during initial stent deployment. There was no stent length foreshortening seen in our patients. There was no incidence of stent fracture during initial procedure or on subsequent catheterizations. One patient with aortic atresia and VSD had stent deformation during subsequent catheterization for retrograde aortic arch balloon angioplasty. The PDA stent needed to be re-dilated in this patient due to the deformation.

Our interstage mortality rate of 12% is on the lower end of the spectrum when compared to published literature [15, 21]. In our cohort, 35% of patients (n = 6) required reintervention, which is comparable when compared to other studies using balloon-expandable stents. Indeed, Gorecezny et al. reported a reintervention rate of 48% using Palmaz Genesis or Palmaz Blue stents (Cordis, Johnson and Johnson, Miami Lakes, Florida, USA)[21]. The rate of reintervention is more frequent when compared to studies using self-expandable stents (18%) reported by Goreczny et al. [9] and (11%) reported by Baba et al.[22]. Part of the reintervention in the interstage period in our cohort can be attributed to having more patients with retrograde aortic arch obstruction, which is a common complication reported in up to 29% of patients during ductal stenting [23, 24]. We did not have angiographic findings of in-stent stenosis due to neo-intimal proliferation as seen in other studies [6, 25].

The removal of the Valeo stent during the next stage of surgical repair was not difficult. In most cases, the ductus was divided and the Valeo stent was removed in its entirety. In three patients, a small portion of the Valeo stent was retained because the stent remained deeply embedded in the proximal descending aorta. There was no incidence of post-operative aortic obstruction in patients who underwent aortic arch reconstruction after the Valeo stent removal.

## Limitations

The small size of our cohort limits our ability to do any meaningful statistical analysis. This is also a single center study that is reliant on our local institutional experience, which limits its generalizability. We are unable to directly compare the Valeo stent with other stent types such as self-expandable stents or a different balloon-expandable stents as we did not incorporate them into our practice.

## Conclusion

Our experience with the Valeo stent demonstrates that it is a feasible option for ductal stenting in hybrid palliation. We experienced a low complication rate, a low interstage mortality rate, and low rates of reintervention. Studies that compare the performance of other balloon-expandable stents and self-expandable stents in hybrid palliation will be helpful to guide the interventional community regarding their safe use.

## Data Availability

No datasets were generated or analysed during the current study.
